# Age and professional experience as determinants of the utilization of psychoneuroimmunological research in clinical practice: An exploratory study

**DOI:** 10.1097/MD.0000000000034723

**Published:** 2023-08-25

**Authors:** Christian Mareth, Ulrich K. Fetzner, Christoph H. Saely

**Affiliations:** a UFL Private University in the Principality of Liechtenstein, Triesen, Principality of Liechtenstein; b AllDent Zahnzentrum, München, Germany; c Department for General-, Visceral-, Thoracic-, Pediatric- and Endocrine Surgery, Johannes Wesling Hospital, University Clinic Ruhr, University Bochum, Minden, Germany; d Vorarlberg Institute for Vascular Investigation and Treatment (VIVIT), Feldkirch, Austria.

**Keywords:** psychoneuroimmunology, inflammation, information dissemination, psychosocial intervention

## Abstract

The immune system is affected by psychosocial stimuli and plays a major role in the development of various diseases. Psychoneuroimmunology (PNI)-based interventions may positively influence the disease course; however, the impact of PNI research findings on clinical practice differs depending on the medical specialties involved. A comprehensive overview of the use of PNI research findings in clinical practice is currently lacking. This exploratory study aimed to provide insight into the dissemination of PNI research findings and their practical applications among clinical practitioners. Data was collected from 50 physicians using an ad hoc online questionnaire. We invited participants to take part in our online survey via an article in the DocCheck Newsletter, a German-language newsletter for physicians. Bivariate nonparametric correlation analysis (Spearman correlation) were used to explore the relationship between independent variables (age, sex, medical specialty, professional experience, and clinical environment) and dependent variables (six questionnaire items concerned with awareness, relevance, and utilization of PNI concepts). While 46% of respondents believed that PNI research findings were relevant to patient treatment, only 22% used PNI-based interventions as part of their therapeutic regimen. Furthermore, 90% of participants could not refer their patients to therapists offering PNI-based interventions. Moderately positive correlations were identified between the increasing age (r_s_ = .48, *P* < .001) and increasing amount of professional experience (r_s_ = .34, *P* = .02) of study participants and awareness of the theoretical foundations of PNI research. Although there is some awareness of PNI among medical practitioners, there appears to be a clear barrier inhibiting the implementation of research findings in current treatment practices. Therefore, it is necessary to examine the impact of increasing age and professional experience on the utilization of PNI-based interventions in patient care.

## 1. Introduction

The immune system is affected by psychosocial stimuli and is a major factor in the development of various diseases,^[1–4]^ including wound healing disorders,^[5]^ viral infections,^[6–8]^ inflammatory diseases,^[9–11]^ and cancer.^[12]^ When inflammation is not resolved, “sickness behavior,” which describes the occurrence of neurovegetative symptoms (e.g., exhaustion and loss of appetite) and neuropsychiatric complaints (e.g., lack of interest and sadness) caused by pro-inflammatory cytokines, may occur.^[13]^

These findings are underscored by psychoneuroimmunology (PNI), a relatively new branch of psychosomatic research. PNI investigates the mutual interactions between the central nervous system, endocrine system, immune system, and psychological factors.^[14–16]^ The hypothalamic-pituitary-adrenal axis and the autonomic nervous system are well-known stress-signaling pathways that contribute to immune system dysregulation. Both, the sympathetic-adrenal-medullary and the hypothalamic-pituitary-adrenal axes are activated by stressors, which eventually provoke the release of pituitary and adrenal hormones that modulate immune function. These hormones include adrenocorticotropic hormone, cortisol, epinephrine, and norepinephrine.^[17]^ A stressor can be defined as an event that surpasses a person's perceived ability to cope. Therefore, differences in the extent to which people exhibit a physiological stress response to specific stressors exist between individuals.^[18,19]^

Innate and adaptive immune responses can be dysregulated by chronic stress because it alters the balance between type 1 and type 2 cytokine levels. Thereby, low-grade inflammation can be induced. Chronic inflammation resulting from long-term stress is causally linked to the risk of numerous diseases, including cardiovascular disease, diabetes, infectious diseases, certain cancers, and autoimmune diseases. Therefore, interventions addressing stress from a pharmacological, nutritional, physical, and psychosocial perspective are of clinical importance in many medical disciplines.^[20,21]^ Until the 1950s, the mutualistic relationship among these organ systems was largely ignored by the medical profession. However, the discovery that mice subjected to daily avoidance conditioning sessions were less susceptible to anaphylactic shock compared to unstimulated controls and evidence of adrenal involvement in mediating these effects have led to increased research interest in the possible interactions among these organ systems.^[22,23]^ The work of Solomon and Moos in the 1960s on the relationship between personality and the presence of the rheumatoid factors^[24]^ and the work of Ader and Cohen in the 1970s on immune system conditioning,^[25,26]^ have been additional milestones in the development of this new field of research.

PNI is a relatively active area of research. A search for the term “PNI” in PubMed in July 2023 identified 3067 publications. Of these, 120 were published in the past 12 months. Clinical studies have shown that PNI-based interventions can positively influence the course of various diseases. For example, a mindfulness-based intervention effectively reduced stress and pro-inflammatory signaling in younger (age 28–60) breast cancer survivors.^[27]^ PNI-based interventions refer to interventions with proven impact on psychological parameters, as well as neuroendocrine and immunological parameters. The wide range of available interventions includes psychological (psychotherapy and/or counseling), psychosocial (relaxation, meditation, mindfulness-based stress reduction, and/or cognitive-behavioral stress management), physical (e.g., yoga, taijiquan and/or qigong), and pharmacological interventions.^[28,29]^

However, the impact of these findings on patient care differs across medical specialties. Although PNI-based interventions are a standard component of quality cancer care,^[30]^ limited information is available on the adoption of these interventions in other medical specialties, including primary care. The Institute of Medicine (U.S.) noted in 2001 that a substantial gap existed between the care that could be delivered if health care professionals were informed by scientific knowledge and the care that is delivered in practice.^[31]^ Implementing prevention and treatment strategies that align with current evidence-based knowledge can preclude avoidable morbidity, mortality, and healthcare expenditures.^[32]^ Currently, a cohesive overview of the utilization of PNI research findings in clinical practice is lacking. This exploratory study aimed to identify parameters influencing the use of PNI-based interventions in clinical practice.

## 2. Methods

### 2.1. Study design

An exploratory study was conducted to identify parameters influencing the use of PNI-based interventions in clinical practice. An online questionnaire was administered to practicing physicians to explore their current level of knowledge on PNI research and the use of insights from this research in clinical practice. The questionnaire was provided in German and English and developed in 2 phases, wherein the limitations of the test construct and generation of indicators followed an experience-based-intuitive approach.^[33]^ Initially, a focus group of 4 clinically active physicians identified indicators needed to answer the research question. The focus group identified the following indicators: awareness of PNI research findings (awareness); assessment of the relevance of PNI research findings to the medical work of the practitioner (relevance); and the degree to which PNI interventions were used in the context of the practitioner medical practice (utilization). For each of the three indicators, 2 questions were developed and evaluated using a 5-point Likert scale.^[34]^ Awareness was addressed by questions 1 and 2, relevance by questions 3 and 4, and utilization by questions 5 and 6 in the mandatory survey (Table 1). The answers to these questions were weighted from 1 point (no approval) to 5 points (full agreement).

**Table 1:** Online questionnaire in English.

1) How well are you aware of the theoretical foundations of psychoneuroimmunological (PNI) research?				
Not at all	Slightly	Somewhat	Moderately	Extremely
2) How well are you aware of the practical applications of PNI research?				
Not at all	Slightly	Somewhat	Moderately	Extremely
3) How likely is it that you will continue to engage with the findings of PNI research?				
Extremely unlikely	Unlikely	Neutral	Likely	Extremely likely
4) PNI interventions are relevant for the therapy of my patients. How much do you agree with this statement?				
Strongly disagree	Disagree	Neither agree nor disagree	Agree	Strongly agree
5) I regularly recommend PNI interventions to my patients as part of their therapy. How much do you agree with this statement?				
Strongly disagree	Disagree	Neither agree nor disagree	Agree	Strongly agree
6) I’m aware of therapists offering PNI interventions in the catchment area of my practice. How much do you agree with this statement?				
Strongly disagree	Disagree	Neither agree nor disagree	Agree	Strongly agree
7) If PNI interventions are already part of your therapeutic regime, which interventions do you recommend to your patients on a regular basis?				
8) If PNI interventions are already part of your therapeutic regime, for which diseases/complaints do you recommend PNI interventions?				
9) If you rate PNI concepts as irrelevant to your work, what reasons have led to this assessment?				
10) Are you male or female?				
11) How old are you?				
12) How many years have you already worked as a medical doctor?				
13) In which medical specialty are you predominantly active?				
14) In which clinical environment are you mainly active in the medical field? (private practice, hospital)				

The 11 mandatory questions in the questionnaire also comprised 5 socio-demographic questions (sex, age, professional experience [years], medical specialty, and clinical environment [private practice or hospital]). Three free-text questions (questions 7–9) were optional. The free-text questions asked for details on PNI-based interventions that were already part of the respondents’ therapeutic regimen, the circumstances (complaints or diseases) under which these interventions were suggested to their patients, or the reasons for the irrelevance of PNI-based interventions in their work as physicians.

### 2.2. Study population

To obtain a comprehensive overview of the dissemination and application of findings from PNI research, the involvement of different medical disciplines was necessary. Therefore, professional activity as a medical doctor was the only inclusion criterion in our study.

We invited participants to take part in our online survey through an article published in the DocCheck Newsletter, a German-language newsletter for physicians. This newsletter is sent periodically to several thousand medical professionals. The online questionnaire was available from July 2018 to March 2019.

### 2.3. Statistical analysis

All questionnaires were completed by the participants, and no participants were excluded. The normal distribution of the data was assessed using the Shapiro–Wilk test. Descriptive statistics were used to assess the characteristics and distribution of the variables. Bivariate nonparametric correlation analyses (Spearman correlation) were used to explore the relationship between independent variables (age, sex, medical specialty, professional experience, and clinical environment) and dependent variables (six questionnaire items concerned with awareness, relevance, and utilization of PNI concepts). The sample size for Spearman correlation was determined using a power analysis. Power analysis was conducted using G*Power version 3.1.9.3 (University of Düsseldorf, Düsseldorf, Germany) with an alpha of 0.05, a power of 0.80, and a medium-to-large effect size (0.4) for a 2-tailed test. Since Spearman rank correlation coefficient is computationally identical to the Pearson product-moment coefficient, power analysis was conducted using the G*Power module to estimate the power of Pearson correlation. Based on these assumptions, the required sample size was determined to be 46. Thus, a sample size of N = 50 was adequate for testing the study hypothesis. All calculations and analyses were performed using the IBM SPSS statistics software (version 26.0; IBM Corporation, Armonk, NY). The significance level was set at *α* = 5% (.05); results with a 2-tailed *P < α* were considered statistically significant. The optional free-text questions were evaluated using qualitative content analysis.

## 3. Results

A total of 50 clinical practitioners completed the online questionnaire. The participants were aged between 26 and 77 years (Mean [M] = 50.7, standard deviation [SD] = 12.1). They represented 17 medical specialties. Forty percent of the respondents were active in general or internal medicine. Detailed characteristics of the survey participants are presented in Table 2.

**Table 2:** Characteristics of survey participants.

Characteristics	Categories	Respondents
Sex	Female	25 (50%)
	Male	25 (50%)
Age (yr)	20–29	3 (6%)
	30–39	8 (16%)
	40–49	9 (18%)
	50–59	17 (34%)
	60–69	11 (22%)
	70–79	2 (4%)
Professional experience (yr)	0–9	10 (20%)
	10–19	9 (18%)
	20–29	19 (38%)
	30–39	12 (24%)
Clinical environment	Private practice	29 (58%)
	Hospital	21 (42%)
Medical specialty	General medicine	12 (24%)
	Internal medicine	8 (16%)
	Psychiatry and psychotherapy	5 (10%)
	Child psychiatry and psychotherapy	3 (6%)
	Gynecology and obstetrics	3 (6%)
	Psychosomatic medicine	3 (6%)
	Pediatrics	3 (6%)
	Ear, Nose, and Throat (ENT) medicine	2 (4%)
	Dermatology	2 (4%)
	Psychooncology	2 (4%)
	Anesthesiology	1 (2%)
	Anatomy	1 (2%)
	Occupational medicine	1 (2%)
	General surgery	1 (2%)
	Neurology	1 (2%)
	Naturopathy	1 (2%)
	Public health	1 (2%)

### 3.1. Awareness

Almost half of the study participants (46%) reported having little or no knowledge on the theoretical basis of PNI concepts. Approximately 1-quarter of the study participants (24%) reported a moderate understanding of these theoretical concepts.

Bivariate analyses (Spearman-Rho, r_s_) identified moderately positive as well as weak correlations between age, amount of professional experience of the study participants, and the mandatory survey questions belonging to the awareness domain of our survey (Table 3).

**Table 3:** Correlation of age and amount of professional experience with answers to survey questions.

Question	Age		Experience	
	r_s_*	*P*	r_s_*	*P*
1. How well are you aware of the theoretical foundations of PNI research?	**.48**	<.001	**.34**	**.02**
2. How well are you aware of the practical applications of PNI research?	**.32**	**.02**	.19	.18
3. How likely is it that you will continue to engage with the findings of PNI research?	**.29**	**.04**	**.29**	**.04**
4. PNI interventions are relevant for the therapy of my patients. How much do you agree with this statement?	**.41**	<.001	**.31**	**.03**
5. I regularly recommend PNI interventions to my patients as part of their therapy. How much do you agree with this statement?	**.45**	<.001	**.32**	**.02**
6. I am aware of therapists offering PNI interventions in the catchment area of my practice. How much do you agree with this statement?	.21	.15	.18	.21

* r_s_ = Spearman-Rho.

Significance level (bold entries) was set at *α* = 5% (.05)

### 3.2. Relevance

Of the study participants, 64% indicated that it was likely or extremely likely that they would continue to learn more about PNI and its findings. However, 6% of participants indicated that it was unlikely or extremely unlikely that they would consider PNI concepts in the future.

Of the participants, 46% reported that the findings from PNI research were relevant to the treatment of their patients. These findings were not considered relevant by 22% of the study participants. Almost 1-third (n = 16) of the participants were neutral.

Additionally, moderately positive as well as weak correlations were identified between age, the amount of professional experience of the study participants, and the mandatory survey questions related to relevance (Table 3).

### 3.3. Utilization

Approximately half of the participants (52%) did not use PNI interventions as part of their treatment regimens. In 22% of the study participants, PNI interventions were a regular part of patient treatment. Regarding the actual utilization of PNI interventions, 90% of the participants could not refer their patients to therapists offering PNI interventions. Of all respondents, only 1 physician considered himself in the position to do so.

Moderately positive as well as weak correlations between the age and the amount of professional experience of the study participants were identified for one of the mandatory survey questions related to utilization (Table 3).

### 3.4. Free text questions

Twenty-one participants answered the free-text question (multiple answers possible) about PNI interventions that were regularly recommended to their patients. Relaxation skills (n = 7), autogenic training (n = 5), and psychotherapy (n = 4) were the most frequently used interventions. Table 4 provides an overview of these interventions.

**Table 4:** Psychoneuroimmunology-based interventions regularly recommended by survey participants.

Psychoneuroimmunology-based interventions regularly recommended	Number of respondents
Relaxation skills	7
Autogenic training	5
Psychotherapy	4
Meditation	3
Mindfulness-based stress reduction - MBSR	3
Stress management training	3
Exercise	2
Mindfulness training	2
Yoga	2
Acupuncture	1
Breathing exercises	1
Healthy nutrition	1
Qigong	1

Nineteen participants described the areas of application (diseases and/or complaints) of the PNI interventions (multiple answers possible). Oncological diseases were listed as the most common field of application (n = 5), followed by recurrent or chronic infections (n = 3) and depression (n = 3). Two participants reported fatigue and dysmenorrhea. Table 5 presents a complete overview of these results.

**Table 5:** Areas of application for psychoneuroimmunology-based interventions as mentioned by survey participants.

Areas of application for psychoneuroimmunology-based interventions	Number of respondents
Oncological diseases	5
Recurrent or chronic infections	3
Depression	3
Dysmenorrhea	2
Fatigue	2
Allergy	1
Anxiety disorder	1
Asthma	1
Autoimmune diseases	1
Burnout	1
Chronic pain	1
Chronic pelvic pain	1
Disruptive mood dysregulation disorder	1
Hypersensitivity	1
Hypertension	1
Obesity	1
Overactive bladder	1
Psychosomatic disorder	1
Repeated abortion	1
Rheumatoid arthritis	1
Sarcoidosis	1
Somatic syndrome	1
Stress	1
Tinnitus	1
Vertigo	1

## 4. Discussion

The results of this study highlight the limited utilization of PNI-based interventions in clinical practice. While 46% of respondents believed that PNI research findings were relevant to patient treatment, only 22% used PNI-based interventions as part of their therapeutic regimen. This corresponds to the respondents’ assessment of their existing expertise in the field of PNI. Although more than half of the study participants reported having a basic understanding of the principles underlying PNI, their knowledge of the practical applications of PNI-based interventions was less pronounced.

The experience gained during the coronavirus-disease 19 (COVID-19) pandemic has reinforced the importance of applying PNI research findings to clinical practice. The fear associated with the consequences of the COVID-19 pandemic has led to a global increase in psychological distress. Additionally, COVID-19 is associated with activated immune-inflammatory pathways and cytokine storms. Major psychiatric disorders are associated with activated immune-inflammatory pathways in at least a subset of individuals. Therefore, immune-inflammatory processes may be involved in the manifestation of neuropsychiatric symptoms in individuals infected with COVID-19.^[35]^

Both the age of a physician and their years of professional experience appear to affect the awareness and utilization of PNI-based interventions in clinical practice. The reasons for this relationship could not be determined from our data. Xu et al^[36]^ also identified age and experience as relevant factors when they evaluated health professionals’ engagement with the core competencies defined by the Institute of Medicine (U.S.). However, they found that 18 to 34-year-old health professionals were more likely to provide patient-centered care than their 35 to 65+-year counterparts. This seems to contradict our findings but might be explained by differing viewpoints among health professionals with respect to patient-centered care.^[37]^

Age and professional experience have been linked to healthcare quality in various studies. Riordan et al^[38]^ conducted a systematic review to evaluate physician and practice characteristics with respect to the quality of diabetes management in primary care. They identified no association between physician experience and the quality of diabetes care. However, they associated increasing physician age with lower quality of diabetes care. Anderson et al^[39]^ analyzed the association of surgeon age with congenital heart surgery outcomes. According to their study, the odds of major morbidity/mortality were approximately 25% higher in surgeries performed by senior surgeons. Choudhry et al^[40]^ conducted a systematic review to evaluate the relationship between clinical experience and quality of health care. They concluded that physicians may be at risk of providing lower-quality care with increasing professional experience. Although our findings suggest that the increasing age and professional experience of physicians are associated with a greater likelihood of integrating PNI-based interventions into patient care, these factors also appear to have negative effects on the quality of patient care.

The debate about replicability, and therefore, the robustness of experimental study results in psychology, began from 2010 to 2012.^[41]^ This debate also influences the transfer of experimental study results into clinical practice. Efforts to increase the replicability of results in PNI research have led to an intensive discussion on the methods utilized in this research. Moriarity and Alloy^[42]^ argue that case-control studies and total symptom score methods are not sufficient to maximize the understanding of the association between the immune system and psychopathology. Moriarity^[43]^ also argued that improved statistical rigor in PNI research might help produce more replicable and clinically relevant studies. Tresker^[44]^ proposed that PNI research that aims to evaluate the effectiveness of therapeutic interventions based on PNI needs to examine the mechanisms of action, potential adverse effects, and the impact of the placebo effect in these intervention studies. The approaches described above have the potential to improve the robustness of PNI-based intervention studies, and consistent enhancement of the methods utilized in these intervention studies may eventually contribute to the transfer of such study results into clinical practice.

A limitation of this exploratory study is that we must assume that willingness to participate was higher among physicians who already had some exposure to PNI. This assumption is supported by the disproportionate involvement of psychiatry and psychosomatic medicine specialists in our survey. In addition, 40% of our study participants were engaged in general practice or internal medicine. Therefore, our insights can only be transferred to the medical profession to a limited extent. However, this could also indicate that the level of understanding of the principles and applications of findings from PNI research in the medical profession was lower than reported herein. The questionnaires were provided in German and English. However, none of the participants used the English version. The fact that only German-speaking physicians participated in the online survey is another limitation. An important strength of our study was the meticulous characterization of the participating physicians. Thus, we identified the factors that influence the utilization of PNI research findings in the medical profession.

Due to the moderately sized and possibly incompletely generalizable sample of this exploratory study, our preliminary findings must be further assessed in comprehensive studies. In particular, the influence of age and the amount of professional experience on the utilization of PNI research findings needs further investigation. This is particularly relevant, considering that these determinants are often reported to negatively impact the quality of patient care.

Although our survey was completed by clinical practitioners from 17 different medical specialties, we cannot comment on the utilization of PNI research findings within individual medical specialties because of the small sample size. Therefore, the survey should be extended to a larger number of participants including English speaking practitioners.

## 5. Conclusions

This exploratory study suggests that the use of PNI-based interventions in clinical practice is limited and that age and professional experience serve as determinants for the utilization of psychoneuroimmunological research in clinical practice. While there is some awareness of PNI among medical practitioners, there appears to be a clear barrier that inhibits the implementation of research findings in current treatment practices. Therefore, it is necessary to further examine the impact of increasing age and professional experience on the utilization of PNI-based interventions in patient care.

## Acknowledgments

We would like to thank Erik Kirst from PHIMEA (www.phimea.de) for statistical consulting.

## Author contributions

**Conceptualization:** Christian Mareth, Christoph H. Saely.

**Data curation:** Christian Mareth, Ulrich K. Fetzner.

**Formal analysis:** Christian Mareth.

**Investigation:** Christian Mareth.

**Methodology:** Christian Mareth.

**Resources:** Christoph H. Saely.

**Supervision:** Christoph H. Saely.

**Validation:** Christian Mareth, Ulrich K. Fetzner, Christoph H. Saely.

**Visualization:** Christian Mareth.

**Writing – original draft:** Christian Mareth.

**Writing – review & editing:** Christian Mareth, Ulrich K. Fetzner, Christoph H. Saely.
